# Biosynthesis of Silver, Copper, and Their Bi-metallic Combination of Nanocomposites by *Staphylococcus aureus*: Their Antimicrobial, Anticancer Activity, and Cytotoxicity Effect

**DOI:** 10.1007/s12088-024-01229-2

**Published:** 2024-03-08

**Authors:** Mohsen A. Sayed, Tahany M. A. Abd El-Rahman, H. K. Abdelsalam, Sayed M. S. Abo El-Souad, Rawan Muhammad Shady, Radwa Abdallnasser Amen, Mostafa Ahmed Zaki, Martina Mohsen, Sara Desouky, Samar Saeed, Seif Omar, Asmaa A. H. El-Bassuony

**Affiliations:** 1 Botany and Microbiology Department, Faculty of Science, Cairo University, Giza, Egypt; 2 Basic Science Department, Higher Institute of Applied Arts 5th Settlement, New Cairo, Egypt; 3 Biotechnology Department, Faculty of Science, Cairo University, Giza, Egypt; 4 Physics Department, Faculty of Science, Cairo University, Giza, Egypt; 5 Central Lab for Food Safey, School of Veterinary Medicine, Badr University, Cairo, Egypt

**Keywords:** Bi-metallic nanoparticles, *S. aureus*, Cytotoxicity, Antimicrobial, Anticancer activity

## Abstract

The present study outlines an easy, cheap, and environmentally friendly way to make *Staphylococcus aureus*-mediated bimetallic silver-copper nanocomposites (Ag/Cu) that can fight cancer and germs. The gram-positive *S. aureus* synthesized Ag, Cu, and their bi-metallic nanocomposites extracellularly. We aimed to prepare the bimetallic nanocomposite in two different ways, and we compared them in terms of characterization and biological applications. The first one is a bimetallic nanocomposite (Ag/Cu_b_) that was made by mixing Ag and Cu metal ions in equal amounts (50:50). Then, the whole mixture was reduced. The second is the after-reduction bimetallic nanocomposite (Ag/Cu_a_), in which each metal ion was reduced separately, and then the nanocomposites were mixed (50:50%) during biological applications. Nanocomposites were characterized using UV–visible spectrophotometry, Fourier-transform infrared spectroscopy, dynamic light scattering, and transmission electron microscopy. The results demonstrated that surface plasmon bands were at 320 nm for Ag NPs and 525 nm for Cu NPs, and a shift from these peaks was observed at 290 nm in the Ag/Cu_b_ bimetallic nanocomposite. The synthesized nanocomposites were confirmed to be in the nanoscale with 20, 40, and 80 nm spherical crystals, respectively. Nanocomposites were assayed for their antimicrobial activity against the gram-negative *Pseudomonas aeruginosa*, the acid-fast *Mycobacterium smegmatis*, the gram-positive *Bacillus cereus,* and *S. aureus,* in addition to three fungal species, which were *Aspergillus flavus*, *A. fumigatus,* and *Candida albicans*. The minimum inhibitory concentration and minimum bactericidal concentration were determined. The Ag/Cua/Cuaetallic nanocomposite was the most potent antimicrobial compound. The anticancer activity of the tested compounds was assayed against the hepatocellular carcinoma cell line (HepG-2). Low cytotoxic activity was recorded in most assayed nanocomposites against the baby hamster kidney cell line (BHK).

**Supplementary Information:**

The online version contains supplementary material available at 10.1007/s12088-024-01229-2.

## Introduction

Nanobiotechnology emerged as a scientific discipline for novel materials and applications at the turn of the century. Nanoparticles (NPs) are quickly becoming the essential building blocks of nanotechnology. Their modest dimensions and high surface area-to-volume ratio allow them to display unique chemical and physical properties. As a result, nanoparticles can be employed in applications other than those for which they were designed [1–5]. Nanoparticles have electrical resistivity, conductivity, chemical resistance, mechanical strength, responsiveness, and complex and adaptable biological processes [6–10].

Nanomaterials are therapeutic tools in clinical applications [11–15]. Nanoparticles are created in various ways, including through physical, chemical, and biological techniques. The employment of^3^ microorganisms is an enzymatic process among these approaches [16]. These eco-friendly green approaches avoid the use of costly chemicals. Nanoparticles formed by microorganisms collect metal ions from their surroundings and transform them into elemental metals via enzymes produced by these microorganisms [17]. The microorganism-mediated synthesis process can be divided into intracellular and extracellular [18]. The transfer of metal ions into the cell is called intracellular synthesis. Extracellular production of nanoparticles is more prevalent, resulting in a simple and speedy reaction. Enzymes and proteins in the culture filtrate come into direct contact with heavy metals during this process. As a result, nanoparticles are made very quickly [19].

Nanobiotechnology can solve two global issues. Firstly, antimicrobial resistance. Microbes become resistant to commercial antimicrobial drugs after long-term use in illness treatment; as a result, there is high anticipation and an urgent need for medicines with unique antimicrobial capabilities [20].

Secondly, cancer is the primary cause of disease-related mortality and aberrant cell and tissue proliferation. Cancer is still one of the most severe diseases in the world, and treatment includes surgery, radiation, and chemotherapy medications, which frequently kill healthy cells and cause toxicity in humans. Because of the introduction of nanotechnology, drug design, and cancer imaging are rapidly progressing [21]. Cell lines and culture conditions for in vitro research were used to assess nanoparticle anticancer efficacy.

Because of their distinctive physical and chemical properties, silver nanoparticles (Ag NPs) are rapidly used in various industries, including food, health care, and medicine [22–25]. These include optical, electrical, and thermal properties, strong electrical conductivity, and biological qualities [26–28].

Silver ions are reduced by biomolecules such as amino acids, proteins, NAD+ reductases, dehydrogenases, and different secondary metabolites [29]. Extracellular proteins, enzymes, and peptides are capping agents [30]. The capping agents stick to the surface of the biosilver nanoparticles and keep them stable. They stop the nanoparticles from sticking together and change their shape by stopping them from growing out of control [31].

Cu NPs green synthesis is environmentally benign, economically practical, and does not require harmful chemicals, making Cu NPs appealing for biological applications [32]. Cu NPs also produce reactive oxygen species, which are toxic at high concentrations and inhibit microorganism development. Copper nanoparticles have a variety of applications, including antibacterial, antifungal, antiviral, anticancer, and antioxidant, and are used in drug delivery [33]**.**

The bimetallic synthesis of Ag and Cu prevents Cu metal oxidation. It helps stabilize the system in an open atmosphere [34], making it cost-effective and eco-friendly. Biological reduction in Ag/Cu bimetallic synthesis is another excellent and advantageous way of producing high-purity, nano-sized, and uniform-shaped nanoparticles [35]. Bimetallic nanoparticles (composition of two different metals to make one composite or alloy) have recently received more attention than individual metallic nanoparticles due to their superior thermal, catalytic, and therapeutic capabilities [36–39].

We aimed to use *S. aureus* as a fast-growing strain; it also confirmed its ability through literature to synthesize nanoparticles extracellularly [40]. The present study has focused on the development of an extracellular biosynthesis of Ag, Cu nanocomposites, and Ag/Cu_a_ and Ag/Cu_b_ bimetallic nanocomposites using *S. aureus* ATCC 6538 cell-free filtrate to provide promising antimicrobial and anticancer compounds. The ultrastructure observation was used to investigate the synthesis location of the *S. aureus*-based nanocomposites. After metal ions were subjected to *S. aureus* cells, scanning electron microscopy (SEM) and transmission electron microscopy (TEM) examinations were carried out. Most of the nanocomposites were found on the surface of the *S. aureus* cells, indicating that the nanocomposites were employed to synthesize outside of the cells. Thus, *S. aureus* captured Ag^+^ and Cu^+^ from AgNO_3_ and CuSO_4_ solutions and bio-reduced them into Ag NPs and Cu NPs extracellularly, and *S. aureus* metal ion reductase was also present extracellularly [41]. The primary way that biological nanocomposites are made outside of cells is through nitrate- or NADH-dependent reductase reduction, with a few biosorption and complexation processes being seen [42]. Extracellular synthesis of nanocomposites from microorganisms requires little additional energy input and yields nanocomposites with fewer operations in subsequent processing [43–45].

Our study aimed to compare two bimetallic nanocomposites mixed before and after reduction by studying their characterizations and biological applications. We synthesized Ag NPs and Cu NPs separately and mixed them after reduction (Ag/Cu_a_) (50:50%) once they were investigated in biological studies. Meanwhile, we synthesized the bimetallic nanocomposite (Ag/Cu_b_). We mixed (50:50%) the Ag^+^ and Cu^+^. Their solutions were subjected to the filtrate, and the bimetallic combination (Ag/Cu_b_) was reduced as one whole. Biosynthesized nanocomposites were characterized using DLS, TEM, FT-IR, and UV–VIS spectroscopy. The antimicrobial activity of nanocomposites was evaluated against selected Gram-positive (*S. aureus* and *B. cereus*), Gram-negative (*P. aeruginosa*), acid-fast bacteria (*M. smegmatis*), and some fungal species (*Aspergillus flavus, A. fumigatus, and Candida albicans*). The anticancer activity was carried out against the human cancer liver cell line (HepG2) and baby hamster kidney (BHK) cell lines via an MTT assay.

## Material and Methods

### Biosynthesis of Ag NPs, Cu NPs, and the Bimetallic Nanocomposite (Ag/Cu) from *S. aureus*

*S. aureus *was cultured in 100 ml of nutrient broth medium (NB) in a 250 ml Erlenmeyer flask and incubated at 37 °C in a shaking incubator at 150 rpm for 24 h. Cu NPs were synthesized according to John et al. [31] with a few modifications. Following the incubation, the cell-free filtrate was centrifuged at 7000 rpm at 4 °C for 10 min. For CuSO_4_·5H_2_O 1 mM dissolved in MilliQ water was added to 100 ml of cell-free filtrates to reach an initial concentration of 5 mg/mL, giving it a blue color. This mixture was incubated at 25 °C for 24 to 48 h on a rotatory shaker at 150 rpm, and then this mixture was statically incubated at 37 °C for 24 h. For Ag NPs, 1 mM of AgNO_3_ was dissolved in 5 mL of MilliQ water and added to the cell-free filtrate to reach an initial concentration of 5 mg/ml [46]. The solution was incubated at room temperature in light and static conditions for 24 h. The color of the Ag NPs solution changed from yellow to dark brown, then was centrifuged and sent for further characterization [47]. Concerning Ag/Cu_a_, the separately synthesized Ag NPs and Cu NPs were mixed while loading in a ratio of 50:50% to produce Ag/Cu_a_. The mixture of CuSO_4_.5H_2_O and AgNO_3_ was dissolved in MilliQ water until it reached a final concentration of 1 mM. Then, 10 ml of the filtrate was added to make the mixture less dense. The mixture was incubated under static conditions at room temperature for 24 h. The nanocomposites produced were centrifuged at 10,000 rpm for 10 min. Then, they were washed with deionized water and centrifuged three times.

### Characterization of the Biosynthesized Nanocomposites

The biosynthesized NPs (Ag, Cu, and Ag/Cu_b_) were assayed by ultraviolet spectra (UV) to analyze the color change of solutions. The spectrophotometer scans from 200–800 nm using Shimadzu UV-1800. The blank was distilled water. We used NICOMP analysis to do dynamic light scattering (DLS) to find out about the solution's Cu NPs, Ag NPs, and Ag/Cu_b_ size. The samples were prepared by re-suspension 320µ NPs in 2 ml distilled water, and then 1.5 ml was transferred to a square cuvette for measurements. Using a Jasco FTIR 300 E spectrometer, Fourier transform infrared (FT-IR) analysis was used to find the possible biomolecules present in biosynthesized Cu NPs, Ag NPs, and bimetallic Ag/Cu_b_. IR spectra were scanned at a resolution of 4.0 cm in the transmission mode of 400–4000 cm. The investigated samples' transmission electron microscopy (TEM) images were obtained using a JEM-1400 flash TEM operating at 80 kV and with 60,000× magnification power.

### Antimicrobial Activity

Microorganisms were obtained from the microbial resource center (Cairo MIRCIN). Gram-positive *S. aureus* (ATCC6538), Gram-negative *P. aeruginosa* (ATCC10145), acid-fast *M. smegmatis* (ATCC19420), and Gram-positive *B. cereus* (EMCC1080) were sub-cultured on nutrient agar (NA) (HIMEDIA). *A. flavus* (ATCC9643), *A. fumigatus* (EMCC103), and *C. albicans* (EMCC105) were subcultured on Sabouraud Dextrose Agar (SDA) (NEOGEN). The agar disk diffusion method, which follows the guidelines of the Clinical and Laboratory Standard Institute (CLSI), was used to test the antibacterial activity of Cu NPs, Ag NPs, Ag/Cu_a_, and Ag/Cu_b_. [48] against four bacterial species, *P. aeruginosa*, *M. smegmatis*, *B*. *cereus*, and *S. aureus,* with a concentration equivalent to the 0.5 McFarland standard of the tested organism. 10 µl of nanocomposites with a concentration of 5 mg/ml was loaded on sterilized disks on nutrient agar (NA) cultured plates that were incubated at 37 °C for 24 h. After incubation, the inhibition zones were measured in mm. Ampicillin (5 mg/ml) was used as a control.

Concerning antifungal activity, the synthesized nanoparticles were assayed for their antifungal activity against three fungal species: *A. flavus*, *A. fumigatus*, and *C. albicans,* by the disc diffusion method [49] at concentration 1 McFarland on Sabouraud dextrose agar (SDA) media containing discs loaded with NPs. Each disc contained 10 µl of 5 mg/ml NPs, both singly and in combination. The inhibition zones were measured in mm after incubation at 25 °C for 48 h. Tioconazole (5 mg/ml) was used as a control.

### Determination of MIC

The standard broth microdilution method assayed the minimum inhibitory concentration (MIC) test according to CLSI 2012 [50]. In 96-well plates, 100 µl of nutrient broth (NB) medium was added to each well for the MIC test. Nanocomposite solutions were then added. The tested microorganisms' viability was tested at different concentrations ranging from 2500 to 0.2 mg/ml. Finally, 0.5 µg/ml of a bacterial suspension with a concentration equal to the 0.5 McFarland standard was added. The positive control was the bacterial suspension and NB medium, while the negative control was the NB medium only. Then, the MIC was determined after incubation at 37 °C for 24 h. In a clean environment, 0.6 g of resazurin powder was mixed with 1 ml of sterilized distilled water to make a 6 mg/ml stock solution. This was done according to the method described by Prabst et al. [47, 51]. The dye solution was diluted with a ratio of 1:10 and loaded with a volume of 5 µl into each well, then incubated at 37 °C for 24 h in the dark.

To determine MICs of *C. albicans,* a calorimetric method was used with resazurin as an indicator [52]. The plate was incubated at 37 °C for 48 h. The wells, which appear blue, indicate the inhibition activity of the biosynthesized nanocomposites. Meanwhile, the wells, which appear in pink, indicate the growth of the microorganisms.

To calculate the relationship between the assayed nanocomposites as antimicrobial agents and the fractional inhibitor index (FIC), the following equation is used:

1 $$\frac{A}{{MIC}_{A}}+ \frac{B}{{MIC}_{B}}= {FIC}_{A}+ {FIC}_{B}=FIC\,Index$$

where A and B are the MIC of each Ag/Cu_a_ loaded in a single well of the 96-well plate, and MICA and MICB are the MIC of each assayed nanocomposite individually.

This study uses the FIC Index value to measure how well the two nanocomposites loaded together (Ag and Cu_a_) work as antimicrobials [53].

### Determination of MBC

The minimum bactericidal concentration was assayed to determine the minimum concentration that killed the bacterial species. The bacterial suspension in blue-colored wells indicating inhibition activity of the nanocomposites that contain the highest concentrations of Ag NPs, Cu NPs, Ag/Cu_b_, and Ag/Cu_a_ were cultured on nutrient agar plates and incubated at 37 °C for 24 h against 4 bacterial species, which were: *S. aureus, P. aeruginosa*, *M. smegmatis*, and *B*. *cereus*, with a concentration equivalent to 0.5 McFarland standard of the assayed microorganisms. 10 μl of the biosynthesized nanocomposites with a 5 mg/ml concentration were loaded on sterilized discs. The inhibition zones were measured in mm after 24 h, according to CLSI guidelines [50].

### Cytotoxicity Assay

#### Cell Culture and Treatment

The viability test was conducted at the Microanalytical Center, Faculty of Science, Cairo University, on two cell lines: the normal cell line, which was the baby hamster kidney cell line (BHK-21), and the cancer cell line, which is the hepatocarcinoma cell line (HepG-2). BHK-21 and HepG-2 cells were purchased from the National Cancer Institute. Cells were cultured in DMEM. The MTT was determined by the viable cell yield [54]. The optical density was measured at 570 nm, and the cell viability percentage was calculated as [ODS/ODC] × 100, where ODS stands for the sample’s mean optical density. At the same time, ODC is the control’s mean optical density [55].

Cell lines were preserved as “monolayer cultures” using RPMI medium supplemented with 10% FBS and 2% Pen/Strep. Cells were incubated at 37 °C in a water-jacketed incubator at 5% CO_2_ in a high-humidity atmosphere (Thermo Fisher Scientific, USA). The lines were repeatedly sub-cultured to be kept in the exponential growth phase. Sterile conditions were achieved by working under an equipped laminar flow cabinet (Microflow Laminar Flow Cabinet, MDH Limited, Hampshire SP105AA, U.K.). The control and treatment groups comprised cells that were given different amounts of the biosynthesized nanocomposites (12.5, 25, 50, and 100 μg/ml).

#### Cell Proliferation Assay

After 24 h, add 10 μl of the MTT reagent (concentration 0.5 mg/ml) to each well. Incubate the microplate for 4 h. Add 100 μl of the solubilization solution to each well. After complete solubilization of the purple formazan crystals, measure the absorbance of the samples using a microplate (ADX–120 Alta ELISA washer, Germany). The wavelength to measure the absorbance of the formazan product is 570 nm. The cell viability percentage was calculated using the following equation: The cell viability (%) = [ODS/ODC] × 100. ODS is the sample’s mean optical density, and ODC is the control’s mean optical density. The results were displayed by a graph of the percentage of cell viability versus the concentrations of the tested materials using GraphPad Prism 8.0.2. software.

### Statistical Analysis

SPSS software version 22 was used to analyze the data. Data were regularly distributed within groups, according to Kolmogorov-Smirnova and Shapiro–Wilk tests. As a result, parametric analysis was used for data statistical analysis.

## Results and Discussion

### Characterization of the Biosynthesized Nanocomposites

Initially, a change in the color of Ag NPs to dark brown revealed its synthesis, and then after 48 h, a shift in color to dark green indicated the reduction of Cu NPs. Eventually, Ag/Cu_b_ demonstrated a black color in the cell-free filtrate. These results were in authorization with [45, 56–58]. Microbial-based synthesis of nanocomposites and their oxides offers considerable benefits and has attained favor as an alternative to chemical and physical methods [45]. The ionic forms of the metallic salts are exposed to reductase enzymes, produced extracellularly. This enzyme induces an electron shuttle, causing the reduction of ions to nanocomposites [59].

#### UV–VIS Spectrophotometer

The primary standards suggest that the Microbial-based synthesis of NPs is visible observances and surface plasmon resonance (SPR) strength at varied scanning wavelengths (200–800 nm). Ag NPs show maximum absorbance peaks at 320 nm. Commonly, Ag NPs show maximum peaks at ~ 420 nm at pH 8. Nevertheless, it depends on the size and shape frequently found to vary according to the enclosing environment and its interaction with the Ag NPs [60]. Cu NPs show maximum SPR peaks at 518 nm at pH 8. Absorption peaks for Cu NPs have been documented in the 500–600 nm range, thus formulating our result in convenience with Shikha et al. [61]. Eventually, the absorption peak of Ag/Cu_b_ is observed at 290 nm at pH 8. A shift from Ag NPs and Cu NPs peaks is observed in Ag/Cu_b_, suggesting that its size is smaller than Ag NPs and Cu NPs solely, emphasizing the complete reduction of both after 24 h. Mirzaei. M et al. found that the size of NPs may alter their absorbance spectra [62]. Enormous NPs experience localized surface plasmon resonance (LSPR) at longer wavelengths, which explains why Cu NPs have a longer wavelength absorbance peak. In contrast, smaller NPs have more continuous plasmon oscillations, leading the LSPR peak towards shorter wavelengths. This behavior is abundantly seen in the absorbance spectra of Ag/Cu_b_. At short wavelengths, the absorption of incoming light reduces as the radius of the NPs rises. This is because more significant nanoparticles may absorb more incident light. However, the data show that there is an ideal size for NPs. As the NP radius increases, the LSPR peak changes to longer wavelengths, suggesting a shift in the absorbance spectra. The SPR findings are ascribed to the interaction of metal NPs' conduction electrons with incoming light [62].

The energy level at which a material transitions from absorbing to transmitting light is called its band gap. The UV–Vis spectra of Ag/Cu_b_, Ag NPs, and Cu NPs may be utilized to determine the band edge and gap. The absorption spectra generated from UV–Vis spectra offer information on the NPs' light absorption as a function of wavelength. At the same time, the band edge refers to the energy level at which the material transitions from absorbing to transmitting light. The band edge might be connected to the material bandgap. The bandgap is the energy difference between the material's highest occupied energy level (valence band) and its lowest unoccupied energy level (conduction band). The beginning of absorption is the wavelength when absorption begins to grow significantly. This wavelength calculates band edge and bandgap energies [63]. The greatest absorbance peak recorded at distinct wavelengths for various nanoparticles, such as Ag NPs, Cu NPs, and Ag/Cu_b_ NPs, is connected to the materials' band gap and band edge. The band gap energy is calculated using the following equation

2 $$E=\frac{h c}{\lambda }$$

where E represents energy, h is Planck's constant, c is the speed of light, and λ is the wavelength. The greatest absorbance peak of Ag NPs at 320 nm indicates that the band gap energy corresponds to this wavelength. The band gap is the energy difference between the valence band (the highest energy level occupied by electrons) and the conduction band (the lowest unoccupied energy level). The energy associated with the absorbance peak in this example, 320 nm, is proportional to the energy necessary to move an electron from the valence band to the conduction band. Equation (2) may be used to calculate the particular band edge energy. Using Eq. (2), E = 6.21 × 10^–19^ J for Ag NPs' band edge. The band gap of Ag NPs may be calculated using their highest absorbance or SPR peak wavelengths: E = 1.96 eV. The greatest surface plasmon resonance (SPR) peak measured at 518 nm for Cu NPs suggests that conduction electrons collectively oscillate on the nanoparticles' surface. The SPR phenomenon relies greatly on the nanoparticles' size, shape, and composition. When light interacts with nanoparticles at the precise SPR wavelength, resonance develops, causing a considerable increase in light absorption and scattering. The SPR peak wavelength is proportional to the energy needed for collective electron oscillations and may be used to determine the material's band edge. The largest SPR peak at 518 nm indicating that Cu NPs have a band edge is E = 3.82 × 10^–19^ J. The band gap of Cu NPs may be calculated using their highest absorbance or SPR peak wavelengths: E = 2.40 eV. The absorption peak at 290 nm for Ag/Cu_b_ NPs indicates a band gap energy at this wavelength. Ag/Cu_b_ NPs are made up of silver (Ag) and copper nanoparticles. The absorption peak at 290 nm represents the energy necessary for electron transitions in the material. The particular band edge and energy levels would be determined by the composition and arrangement of Ag and Cu in these nanoparticles. The greatest absorbance peaks for certain nanoparticles correlate to wavelengths representing the band gap and band edge energy. Using Eq. (2), E = 6.85 × 10^–19^ J. Using the wavelength, we compute the Ag/Cu_b_ band gap E = 4.29 eV.

The absorbance peaks are described as Ag nanoparticles with their highest absorbance peak at 320 nm, Cu NP's highest SPR peak at 518 nm, and Ag/Cu_b_ at 290 nm. To compute the wavenumber with a wavelength of 290 nm of Ag/Cu_b_, use the following equation:

3 $$Wavenumber \,\left({{\text{cm}}}^{-1}\right)= \frac{1}{Wavelength\, (\upmu {\text{m}})}$$

**Fig. 1:**
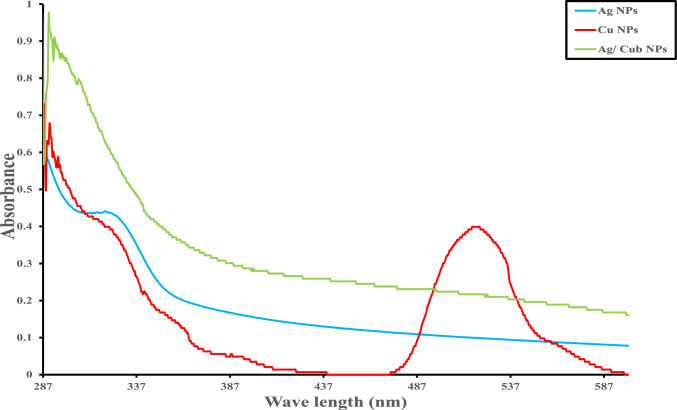
UV–visible spectra of biological Ag NPs, Cu NPs, and Ag/Cu_b_ NPs

When converting the wavelength from nanometers (nm) to micrometers (μm), divide by 1000. The wavelength (μm) may be calculated as 0.29 μm by dividing 290 nm by 1000. The wavelength (cm^−1^) = 1 / 0.29 μm ≈ 3.45 cm^−1^. The wavenumber with a wavelength of 290 nm is about 3.45 cm^−1^. The wavelength (μm) of Ag NPs having a maximal absorbance peak at 320 nm is 0.32 μm, calculated as 320 nm divided by 1000. Using Eq. (3), the wavenumber is calculated as follows: wavelength (cm^−1^) = 1 / 0.32 μm ≈ 3.125 cm^−1^. The wavelength of 320 nm corresponds to roughly 3.125 cm^−1^ for Ag NPs. The wavelength (μm) of Cu NPs having a maximal SPR peak at 518 nm is 0.518 μm when divided by 1000. Using Eq. (3), the wavenumber (cm^−1^) = 1/0.518 μm ≈ 1.931 cm^−1^. Cu NPs have a wavelength of 518 nm, which corresponds to a wavenumber of 1.931 cm^−1^. The maximum absorbance of AgNPs occurs at 320 nm. This suggests that these nanoparticles substantially absorb light in the ultraviolet (UV) range. The particular wavelength of Ag NPs varies based on parameters such as their size, shape, and surrounding environment; Cu NPs have a maximal surface plasmon resonance (SPR) peak at 518 nm. SPR refers to the collective oscillation of conduction electrons in a substance when exposed to light. The SPR peak wavelength is determined by the nanoparticles' size, shape, and composition. In this scenario, Cu NPs resonate and absorb light most efficiently at 518 nm. The absorption peak for Ag/Cu_b_ nanoparticles is at a shorter wavelength of 290 nm. The change in the absorbance peak from Ag NPs (usually approximately 420 nm) and Cu NPs (518 nm) to a shorter wavelength indicates that the Ag/Cu_b_ nanoparticles are smaller than Ag NPs and Cu NPs alone. This shift shows a change in the electrical structure and plasmonic behavior caused by the combination of Ag and Cu. The observed variations in absorption peaks may be ascribed to the nanoparticles' size and composition. Generally, bigger nanoparticles show absorbance peaks at longer wavelengths, but smaller ones move the peak to shorter ones. This phenomenon is referred to as the size-dependent plasmon shift. Interactions with the surrounding environment may also influence the observed alterations. Changes in the electrical structure and resonance behavior of nanoparticles drive these transitions (Fig. 1).

#### Fourier Transform Infrared Spectroscopy (FT-IR)

**Fig. 2:**
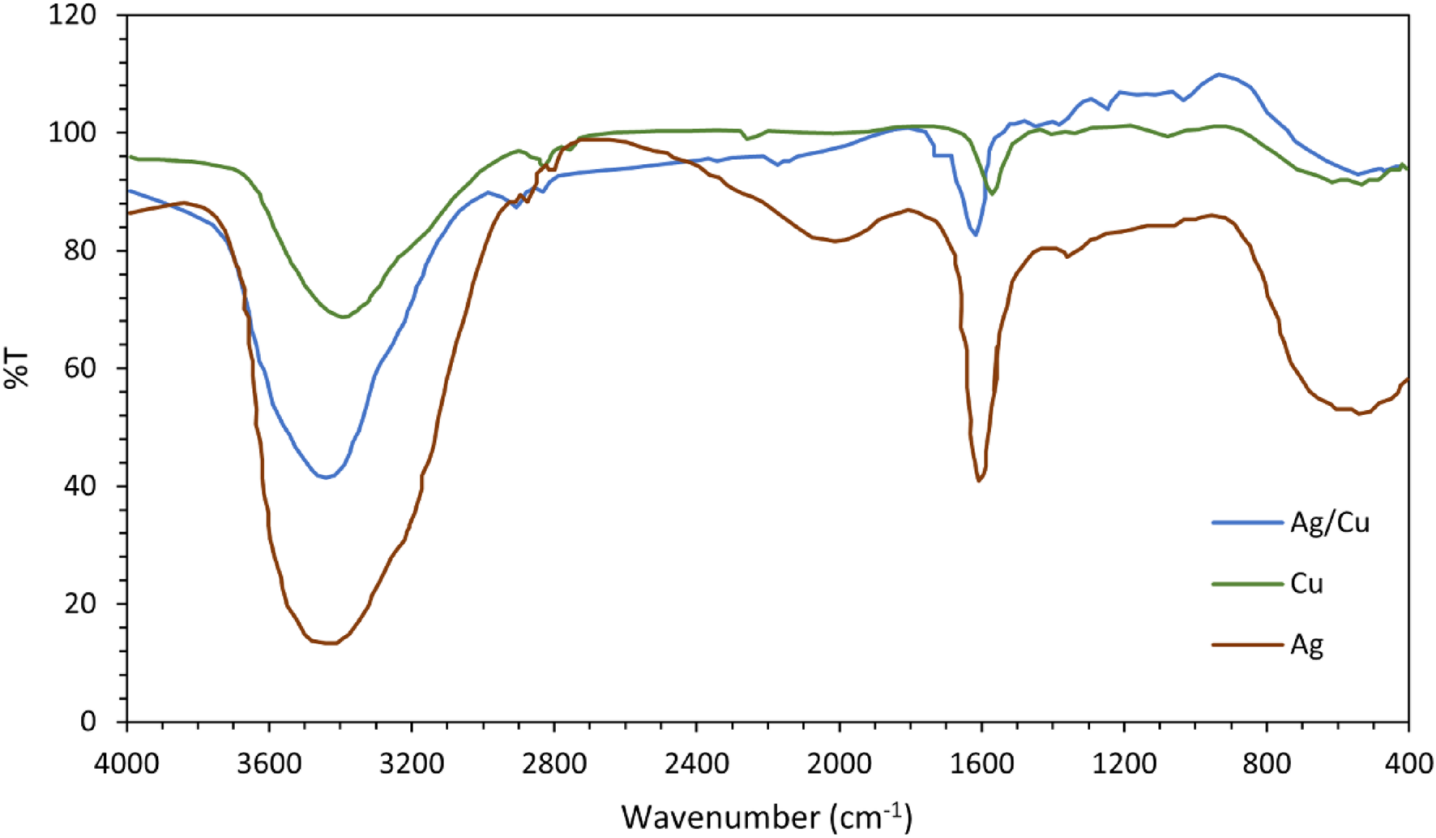
FT-IR spectra of biosynthesized **a** Ag, **b** Cu, and **c** Ag/Cu_b_ NPs

FT-IR analysis was used to confirm the formation of the samples under investigation. The technique known as Fourier Transform Infrared Spectroscopy (FT-IR) creates an infrared absorption spectrum to determine the chemical bonds that are present in a molecule (Table 1 and Fig. 2a–c). FTIR spectra of Ag NPs, Cu NPs, and Ag/Cu_b_ were between 400 and 4000 cm^−1^. Ag/Cu_b_ exhibited more intense peaks than the Ag NPs, and the Ag/Cu_b_ peaks were slightly displaced relative to the Ag NPs' peaks. This is due to the incorporation of diamagnetic material (Cu). The bands around 400 and 500 cm^−1^ were ascribed to the octahedral and tetrahedral sites of the examined samples, respectively. The locations of the absorption peaks at the tetrahedral and octahedral sites are consistent with previous findings [64–66]. The peak at 1030.8 cm^−1^ corresponds to –C–O. The band at 1252.54 cm^−1^ was ascribed to C–O–C stretching vibration. In addition, the absorption bands 1384.6, 1404.9, and 1455.9 cm^−1^ may result from a bonding interaction between O–H and silver nanoparticles. The band at 1637.3 cm^−1^ was attributed to the asymmetric NO_2_ component of nitrate.

**Table 1:** FT-IR showing different peaks stabilized by capping agents in the cell filtrate

No. of Peaks	1	2	3	4	5	6	7	8	9	10	11
Ag	401.2	547.6	–	–	–	1404.9	1637.3	2061.5	–	2926.5	3459.7
Cu	401.1	541.9	1121.4	–	–	1459.9	1637.3	2361.4	2855.1	2923.6	3442.3
Ag–Cu	425.1	538.0	1030.8	1252.5	1384.6	1455.9	1637.3	2192.7	2857.0	2927.4	3454.8

The bands at 2857.0, 2926.5, and 2927.4 cm^−1^ were ascribed to –C–H stretching. Lastly, the 3459.7 and 3454.8 cm^−1^ bands may be attributed to the –OH group. The existence of these functional groups indicates that the components of cell-free filtrate play a dual function, which is utilized as reducing agents and capping agents. Reducing agents aided in the synthesis of nanoparticles via extracellular reduction from ionic to Nano form. They are likewise capping agents, stabilizing nanoparticles and impeding agglomeration and aggregation [67].

#### Dynamic Light Scattering Analysis (DLS)

**Fig. 3:**
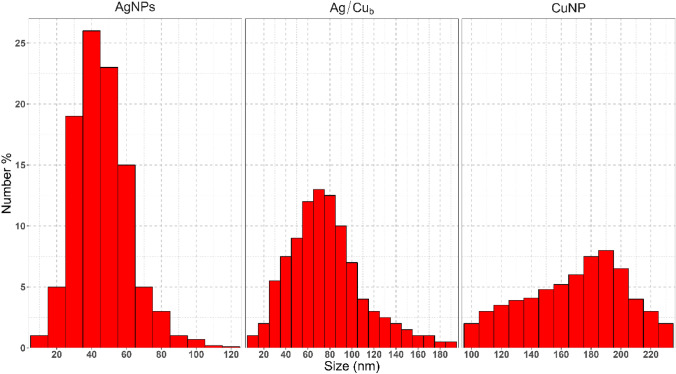
DLS indicates the number-size distribution shows the average size of AgNPs, CuNPs, and Ag/Cu_b_NPs to be 40nm, 180nm, and 80 nm respectively

DLS is a technique for measuring the average particle size and particle size distribution of particles in dispersion based on Brownian motion [68]. The number-size distribution (Fig. 3a–c) shows the average size of Ag, Cu, and Ag/Cu b at 40, 180, and 80 nm, respectively. This examination confirmed that the samples under consideration were nanosized.

#### Transmission Electron Microscopy (TEM)

**Fig. 4:**
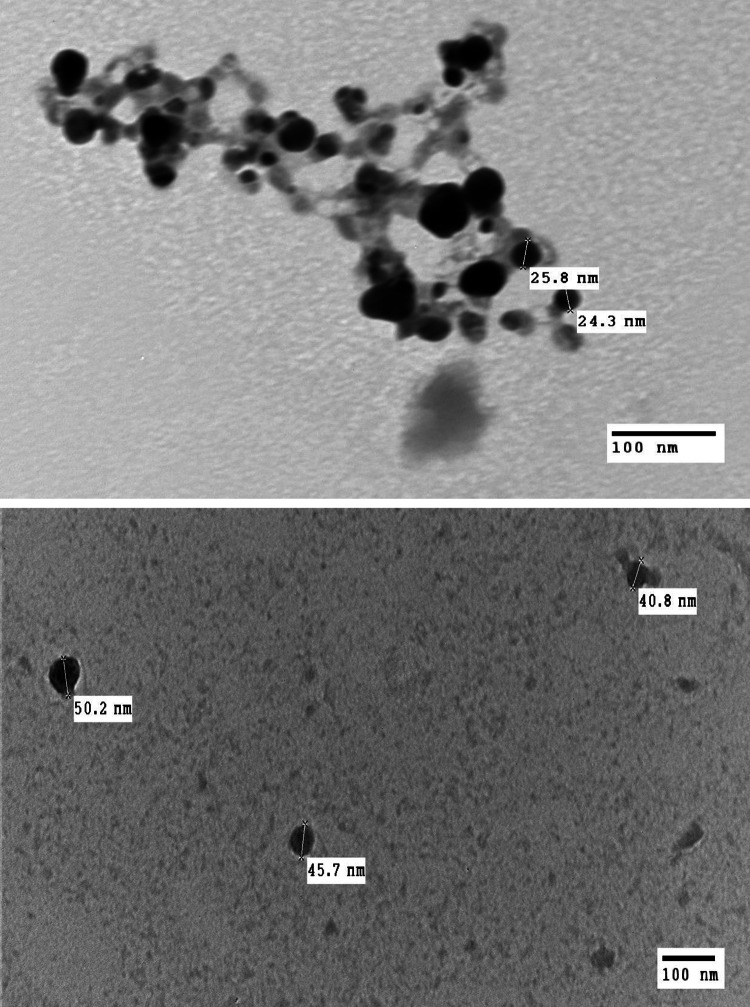
Transmission electron microscope image of produced spherical **a** Ag, **b** Cu nanoparticles using cell-free filtrate of Staphylococcus aureus ATCC 6538

**Fig. 5:**
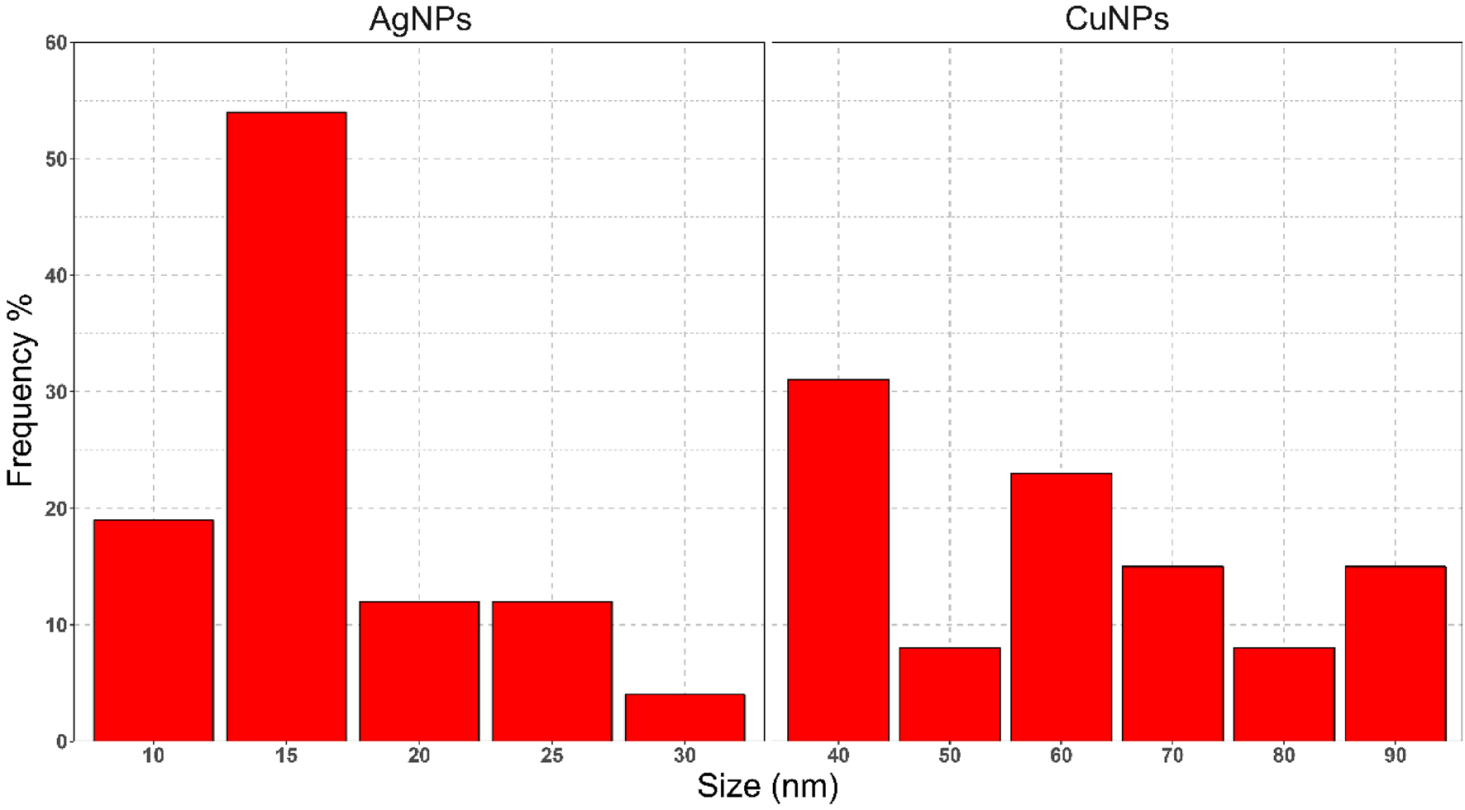
Size distribution of AgNPs and CuNPs obtained from TEM images

TEM analysis of Ag and Cu nanocomposites was displayed (Fig. 4). The Histogram extracted from the images shows that Ag NP size ranges from 11 to 30 nm. The size of the Cu NPs ranges from 40 to 90 nm (Fig. 5). Thus, this is evidence that the investigated samples are in the nanosized range. Moreover, both silver and copper nanocomposites formed as spherical crystals; furthermore, the particles appeared well distributed as each particle can be demonstrated singly, emphasizing the stabilizing capability of the biological capping agents in preventing agglomeration. In DLS data, number-size distribution data is preferred to be used instead of intensity-size distribution since number distribution is less likely to be influenced by other components in the medium, such as dust contaminants than intensity. In addition, it has a better approximation to the TEM size, as Souza et al. mentioned [69].

The size obtained by DLS was 2 to 3 times larger than that obtained from TEM images since DLS measures hydrodynamic sizes of moving nanoparticles within the solution. So, the size can be altered as particles aggregate, overlap, and the formation of an electrical double layer. At the same time, in TEM, by imaging, we can exclude aggregated particles from the analysis [70–72].

### Antibacterial and Antifungal Assays

**Fig. 6:**
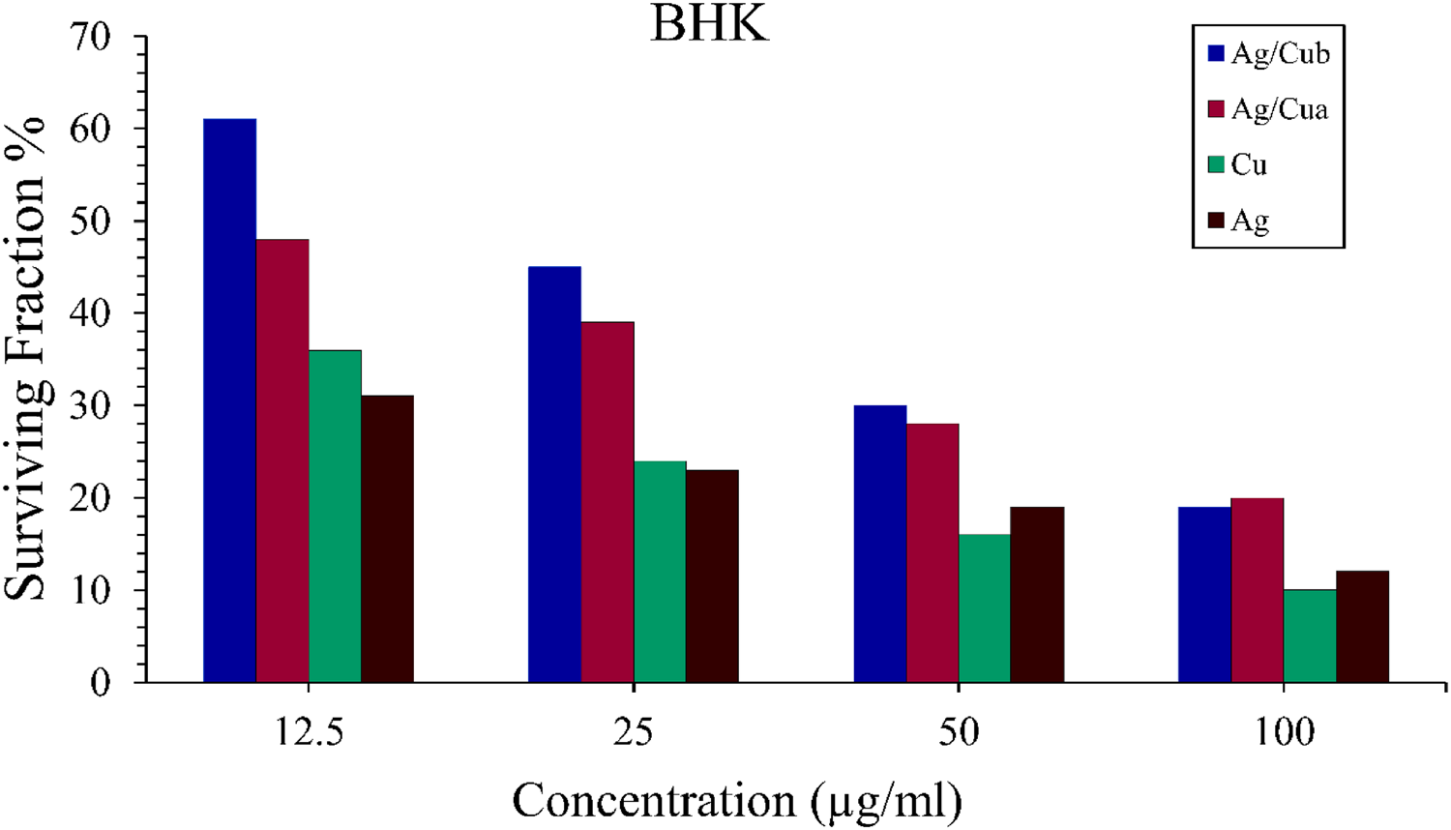
Cell cytotoxic effect of biosynthesized nanoparticles

The bio-synthesized nanocomposites exhibit significant antibacterial activity against all tested bacterial species with inhibition zone diameters ranging from 9 to 30 mm (Table 2 and Fig. 6). Biosynthesized NPs exhibit antimicrobial activity like Ampicillin. The most susceptible microorganism was *P. aeruginosa*, followed by *B. cereus*, then *M. smegmatis,* and *S. aureus*. Ag NPs and Cu NPs showed significant antibacterial activities. Gram-negative bacteria were the most susceptible species to silver nanocomposites. *S. aureus* (Gram-positive) was less susceptible to all assayed nanocomposites. The minimal bactericidal concentration (MBC) was equal to or less than the minimum inhibitory concentration (MIC) results.

**Table 2:** Antibacterial activity of biosynthesized nanoparticles using disc diffusion method

Agent	Inhibition zone diameter (mm)			
	Gram-positive		Acid-fast	Gram-negative
	*B. cereus*	*S. aureus*	*M. smegmatis*	*P. aeruginosa*
Ampicillin	20^b^ ± 0.57	20^c^ ± 0.57	–	–
Ag NPs	24^d^ ± 0.57	22^d^ ± 0.57	20^c^ ± 0.57	30^c^ ± 1.15
Cu NPs	14^a^ ± 1.1	14^a^ ± 0.57	15^a^ ± 0.57	9^a^ ± 0.57
Ag/Cu_b_	20^b^ ± 0.57	15^a^ ± 0.57	16^a^ ± 0.66	23^b^ ± 1.15
Ag/Cu_a_	22^c^ ± 1.0	17^b^ ± 0.57	18^b^ ± 0.57	30^c^ ± 1.15

^a^is the highest significant value

^b^, ^c^are the medium significant value

^d^is the least significant value

Concerning antifungal activity, it was found that the tested nanocomposites did not show antifungal activity against filamentous fungi*. *Only *C. albicans* showed inhibition zone diameters ranging from 10 to 12 mm (Table 3), the same as Tioconazole (control).

**Table 3:** Antifungal activity of biosynthesized nanoparticles using disc diffusion method

Agent	Inhibition zone diameter (mm)
	*C. albicans*
Fungicide	10
Ag NPs	12^a^ ± 1.15
Cu NPs	11^a^ ± 0.57
Ag-Cu_b_	11^a^ ± 0.57
Ag-Cu_a_	10^a^ ± 0.57

^a^is the highest significant value

^b^are the medium significant value

Ag NPs have been shown to have a strong antibacterial effect on both Gram-positive and Gram-negative bacteria. Three mechanisms by which Ag NPs exert their antibacterial action have been seen together or separately. Ag NPs work at the membrane level because they can pass through the outer membrane and accumulate in the inner membrane, where their adhesion causes the cell to become damaged and destabilized. This increases membrane permeability, causes cellular content to leak, and eventually causes the cell to die. Additionally, there is evidence that Ag NPs can interact with sulfur-containing proteins in bacterial cell walls, which may result in structural damage and cell wall rupture. The second mechanism suggests that nanocomposites can enter cells, where it has been suggested that due to Ag NPs' characteristics, they will have an affinity to interact with sulfur or phosphorus groups present in intracellular content such as DNA and proteins, changing their structure and functions. Nanoparticles can also break and cross the cell membrane, altering its structure and permeability. Similar to how they can damage intracellular machinery, activate the apoptosis pathway, and change the respiratory chain in the inner membrane by interacting with thiol groups in the enzymes and generating reactive oxygen species and free radicals, the release of silver ions from the nanoparticles, which because of their size and charge can interact with cellular components and change metabolic pathways, membranes, and even genetic material, is the third mechanism that is hypothesized to happen concurrently with the other two [73].

In addition, due to modifications in membrane shape, Cu NPs enter the bacterial cell and cause cell death by significantly increasing cell permeability and interfering with transport across the plasma membrane. Additionally, various mechanisms underlying Cu NPs' antimicrobial activity have been identified. These include producing reactive oxygen species, protein and lipid oxidation, cell membrane destruction, DNA degradation, and reactive oxygen species [74].

Also, Ag NPs may build up on the bacterial cell walls and membranes, control membrane proteins, and change the membrane's permeability to allow the transit of both Ag NPs and ions into the bacterial cells. After entering cells, Ag NPs continue to produce ions that can damage DNA and proteins. Ag ions generate intracellular ROS, which could impact proteins and DNA; Cu NPs also penetrate bacterial cells and interact with membrane proteins, which causes an upsurge in ROS in the intracellular environment [75].

The thick cell wall of Gram-positive bacteria accounts for the low potency of Ag nanocomposites against *S. aureus* [76]. Molds can evolve more mechanisms of resistance against antifungal agents. In brief, we can conclude that the nanoparticles assessed are significant antibacterial agents rather than antifungal agents [77]. The antimicrobial activity of Ag NPs is expected to be a consequence of reactive oxygen species (ROS), which prompt cell self-destruction [78, 79]. ROS are produced due to the inactivation of the respiratory enzyme chain caused by the dissociation of silver nanoparticles into silver ions. These silver ions also interact with the thiol groups of various enzymes [58].

On the other hand, Copper metal has been authorized and approved as an antimicrobial agent by the United States Environmental Protection Agency. Cu NP's inhibitory action can be attributed to their minor size and elevated volume-to-volume surface area, which entitles them to interact and affect the cell membranes of the microorganisms [80]. Furthermore, their action is associated with the generation of hydroxyl radicals, which destroy the double-stranded DNA as it binds to it and damages essential proteins by binding amino sulfhydryl and carboxyl amino acid groups, thus inactivating crucial enzymes [81]. Cu NPs inhibitory activity is further correlated to an inactivated surface protein. This inactivated surface protein transports the cytoplasmic membrane in addition to selective permeability destruction [60, 82].

Some nanoparticles exhibit antibacterial and antimicrobial properties due to the high redox potential of the surface species created by photoexcitation, allowing for nonselective oxidative attack on bacteria. Photocatalytic inactivation of microorganisms generates reactive oxygen species (ROS) [83]. Ag and Cu nanoparticles demonstrate antibacterial action in a variety of ways. They may induce oxidative stress in bacteria. When exposed to UV radiation, nanoparticles generate reactive oxygen species (ROS) such as hydroxyl radicals, hydrogen peroxide, and superoxide radicals. These ROS may harm bacterial cells and biomolecules. Ag NPs and Cu NPs may release metal ions, such as Ag^+^ and Cu^2+^, which have antibacterial capabilities by interfering with and disrupting bacterial cell membranes.

Furthermore, Ag/Cu_b_ nanoparticles have nonselective antibacterial activity. They may fight various bacteria, including Gram-negative and Gram-positive bacteria, without particular targeting. Because of their nonselective activity, they are effective against a wide range of bacterial strains, lowering the risk of bacterial resistance development. Some nanoparticles have a high redox potential, which rises when photoexcited. This high redox potential enables them to create ROS, which causes oxidative stress in bacteria. The release of metal ions provides Ag NPs and Cu NPs redox properties. The antibacterial effect is caused by redox interactions between these metal ions and bacterial components, which induce cellular damage and surface species when they interact with bacteria. When water is exposed, nanoparticles form surface-bound hydroxyl groups (OH), which may subsequently participate in redox reactions. Ag NPs and Cu NPs can release metal ions that interact with bacterial cells. Surface species significantly impact the antibacterial activity of both kinds of nanoparticles. Ag NPs exhibited potent antimicrobial activity. The bimetallic nanocomposite, where the Ag and Cu nanocomposites were mixed after each was reduced separately (Ag/Cu_a_), exhibited as potent as Ag NPs or higher than it. However, it has half volumes of each Ag NP and Cu NP. The enhancement in the antimicrobial activity of Ag/Cu_a_ is assumed to result from the additive action of the bimetallic nanoparticles on the cell membranes simultaneously. Thus, the microorganisms cannot resist this multi-action appropriately (Tables 4, 5, 6).

**Table 4:** Determination of MIC of the synthesized nanoparticles against bacterial species

	MIC (mg/ml)			
	*B. cereus*	*P. aeruginosa*	*M. smegmatis*	*S. aureus*
Ampicillin	20	-Ve	-Ve	20
Ag NP	20	10	10	20
Cu NP	50	40	50	80
Ag-Cu_b_	20	20	20	50
Ag-Cu_a_	13	7	8	20

**Table 5:** Determination of MIC of the synthesized nanoparticles against *C. albicans*

Agent	MIC (mg/ml)
Fungicide	8
AgNP	8
CuNP	15
Ag-Cu_b_	8
Ag-Cu_a_	8

**Table 6:** Determination of MBC of the synthesized nanoparticles against bacterial species

MBC (mg/ml)	*B. cereus*	*P. aeruginosa*	*M. smegmatis*	*S. aureus*
Ampicillin	20	-Ve	-Ve	20
AgNP	20	39	78	20
CuNP	50	40	50	80
Ag-Cu_b_	20	20	312	450
Ag-Cu_a_	13	39	312	20

This study also compared Ag/Cu_a_ and Ag/Cu_b_ regarding antimicrobial activity. The Ag/Cu_a_ exhibits higher antimicrobial activity than Ag/Cu_b_. It is suggested that when adding both metal ions in the cell-free filtrate, one metal ion can block the reduction of the other metal according to which is reduced first. In this case, Ag ions were reduced before the Cu ions. In other words, Cu ions were partially reduced in the bimetallic nanocomposite (Ag/Cu_b_), at which the two metal ions were mixed at equal ratios and subjected to filtrate as one whole. This resulted in lower antimicrobial activity of (Ag/Cu_b_) than (Ag/Cu_a_).

According to the *FIC* index calculated, results are shown to be additive or indifferent in the case of (Ag/Cu_a_) bimetallic nanocomposite, as the combination of compounds results in an *FIC* index of 0.5–4. FIC index = 0.91, 0.875, 0.96, 1.25, and 0.94 in *B. cereus, P. aeruginosa, M. smegmatis, S. aureus, and C. albicans*, respectively.

### Cytotoxicity Assay

Ag, Cu, and Ag/Cua were confirmed to be promising low cytotoxic agents when tested against average (BHK) cell lines with IC50 values of 191.8, 145.8, and 100.0 µg/ml, respectively. The IC50 of Ag NPs 191 µg/ml was consistent with the IC50 of green synthesized nanoparticles by Arumai Selvan et al. [84] that showed low cytotoxicity on normal human dermal fibroblasts (NHDF) cell lines. Cytotoxicity of Cu NPs (IC50 = 145.8 µg/ml) was consistent with green synthesized nanoparticles from the fungus *Talaromyces pinophilus* showing low cytotoxicity on regular Vero cell line [84]. Thus, the green synthesized nanocomposites from plants or microbes show lower cytotoxicity on regular cell lines. Cytotoxic activity of Ag /Cua, bimetallic nanocomposite with IC50 = 71.2 µg/ml, may be explained by each silver and copper synthesized solely and inducing multi-cytotoxic activities on the BHK cells as Ag NPs and Cu NPs act on the cell at the same time. Although Ag/Cu_b_ displayed lower cytotoxic activity than Ag/Cu_a_. This is due to the partial reduction of both Ag ions and Cu ions when both were added to the same filtrate as Ag ions were reduced before Cu ions, and this resulted in partial reduction of Cu ions to Cu NPs. According to Ioset et al. [85], compounds are considered cytotoxic when their IC50 is less than 90 μg/ml, so the Ag/Cu_a_ bimetallic nanocomposite was found to be toxic.

### Anti-cancer Activity of the Synthesized Nanoparticles

Several pathways can explain why silver nanoparticles have anticancer characteristics. Silver NPs with cancer cells are effective because they exhibit the enhanced permeation and retention effect (EPR), which causes more and more silver nanoparticles to enter the body and accumulate, either killing the cancer cells or preventing their uncontrolled division. Additionally, Ag NPs influence the signaling physiological pathways, causing early apoptosis or slowing the tumor cells' high rate of cell division. Additionally, some studies claim that activating p53, caspase-3, and p-Er K1/2 by silver nanoparticles affects cell division through a series of processes occurring in the cell [86].

Apoptosis Induction in response to Cu NP Treatment, Cell death with cellular, morphological, and biochemical changes results from activating a series of molecular events known as apoptosis, which is regarded as a significant anticancer mechanism. DNA damage and apoptosis/necrosis are linked to oxidative stress, excessive production of ROS/RNS, and cancer cell Sub G1 arrest. Our findings are consistent with earlier research that found that greenly synthesized nanoparticles caused apoptosis to be induced [87].

**Fig. 7:**
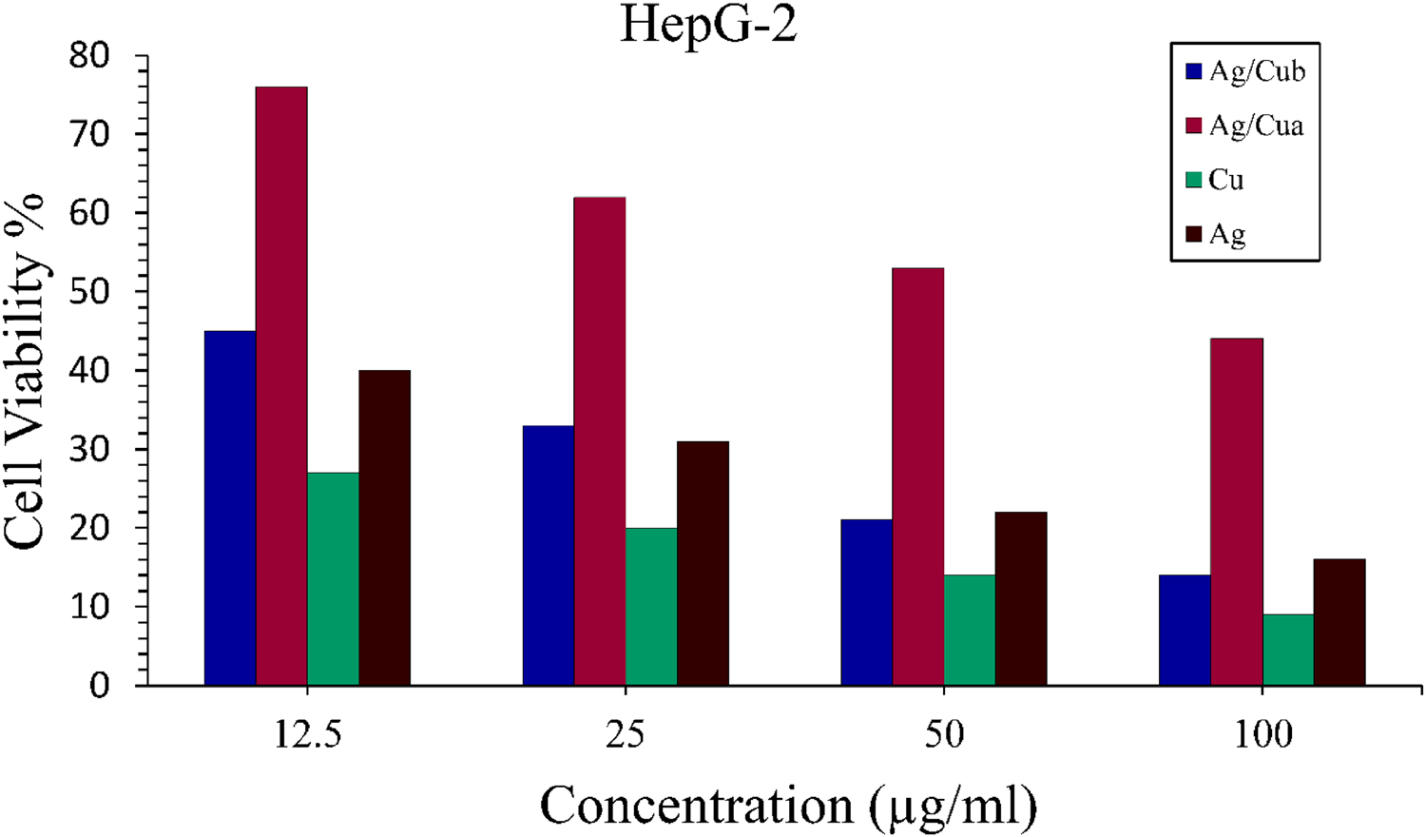
Cell cytotoxic effect of biosynthesized nanoparticles

The MTT test determined the anticancer activity of produced nanoparticles against the Hepatocellular carcinoma cell line (Hepg-2). Cu NPs were shown to be the most promising anticancer agent, followed by Ag NPs and Ag/Cu a with a slight difference. Their IC50 values were determined to be 9 µg/ml, 11 µg/ml, and 11.5 µg/ml, respectively. At the same time, Ag/Cu_b_ had low anticancer efficacy, compared to the positive control, Camptothecin with an IC50 concentration at 2.5 µM equivalent to 13.92 µg/ml used for the study, after the treatment at 37 °C temperature for 24 h of incubation, and negative control of untreated cells (Figs. 6, 7).

**Fig. 8:**
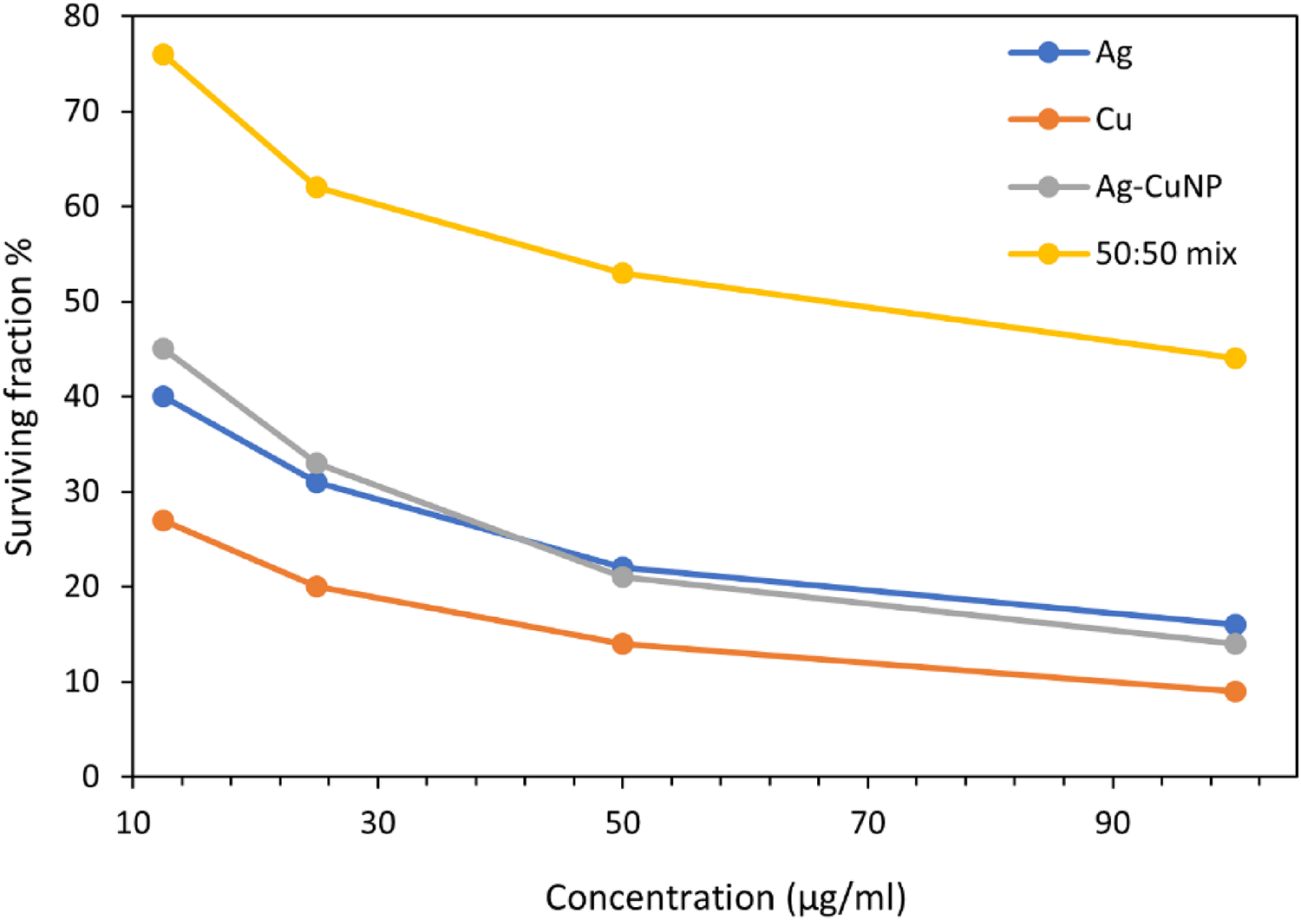
Anticancer activity of biosynthesized nanoparticles

The IC50 value of Cu NPs (IC50 = 9 µg) that were biologically synthesized by *S. aureus* showed promising anticancer activity (Fig. 8), which is consistent with the study of Hassanien et al*.* [87], which showed potent anticancer activity of green synthesized Cu NPs from *Tilia* extract with IC50 = 19.88 µg/ml against Hepg-2 cell line. Another investigation of anticancer activity against the Hepg-2 cell line showed that green-produced Cu NPs from fresh leaves *Azadirachta indica* (Neem) are much higher and more effective than chemically manufactured Cu NPs [45].

According to earlier research [88], Ag NPs with IC50 = 11 µg/ml are likewise considered a potential anti-cancer drug. Because its IC50 value is more effective when compared to Ag NPs produced from *Morus alba* leaf extract with an IC50 value of 20 µg/ml against the same cancer cell line [89]. IC50 value of chemically produced Ag NPs against the Hepg-2 cell line was 75 μg. As a result, microbial-based or green-manufactured nanocomposites (either Cu NPs or Ag NPs) have a better potential to be an effective anti-cancer drug than chemically synthesized nanoparticles [90, 91]. Ag/Cu_a_ also showed intense anti-cancer activity; however, Ag/Cu_b_ had poor anti-cancer activity, which might be attributed to blocking or masking activity at which Ag ions blocked Cu ions during the reduction process.

## Conclusion

In conclusion, *S. aureus* confirmed the ability to synthesize the novel bimetallic (Ag/Cu_b_) nanocomposite and Cu NPs extracellularly. The biosynthesized nanocomposites proved to be on the nanoscale between approximately 20 and 80 nm. They were proved to be stabilized by biological capping agents within the cell-free filtrate. Concerning the antimicrobial activity, the bimetallic nanocomposite (Ag/Cu_a_) has shown enhanced activity, more than the singly loaded ones and (Ag/Cu_b_), due to the additive activity of Ag NPs and Cu NPs. Regarding the comparison between (Ag/Cu_a_) and (Ag/Cu_b_), (Ag/Cu_a_) has better biological activity than (Ag/Cu_b_). This is discussed due to the masking activity of Ag ions that have masked the complete reduction of Cu ions when both are subjected to the same filtrate. On the other hand, (Ag/Cu_a_) were mixed after the reduction of each separately; this ensured the complete reduction of ions to nanocomposites. Concerning the sensitivity of tested microorganisms to nanocomposites, Gram-negative > Gram-positive > Acid-fast bacteria. Regarding the anticancer and cytotoxicity assays. Ag/Cu_b_ and Ag/Cu_a_ bimetallic nanocomposites showed moderate anticancer activity. Ag/Cu_a_ had higher anticancer activity and was relatively more toxic than Ag/Cu_b_ due to the additive effect of each nanocomposite on the cells. At the same time, NPs in (Ag/Cu_b_) were partially reduced. Finally, it is concluded that the biosynthesized nanocomposites proved to be promising antimicrobial and anticancer agents. Thus, they are recommended for further biomedical applications.

## Supplementary Information

Below is the link to the electronic supplementary material.

Supplementary file1 (DOCX 284 kb)

Supplementary file2 (DOCX 393 kb)

Supplementary file3 (DOCX 510 kb)

Supplementary file4 (DOCX 282 kb)

Supplementary file5 (DOCX 542 kb)

## Data Availability

The data that support the findings of this study are available on request from the corresponding author.
