# Supplementary figures and images for: Biosynthesis of Silver, Copper, and Their Bi-metallic Combination of Nanocomposites by Staphylococcus aureus: Their Antimicrobial, Anticancer Activity, and Cytotoxicity Effect
Source: Indian J Microbiol. 2024 Mar 8;64(4):1721–37. doi: 10.1007/s12088-024-01229-2 (PMC11645382; doi:10.1007/s12088-024-01229-2)

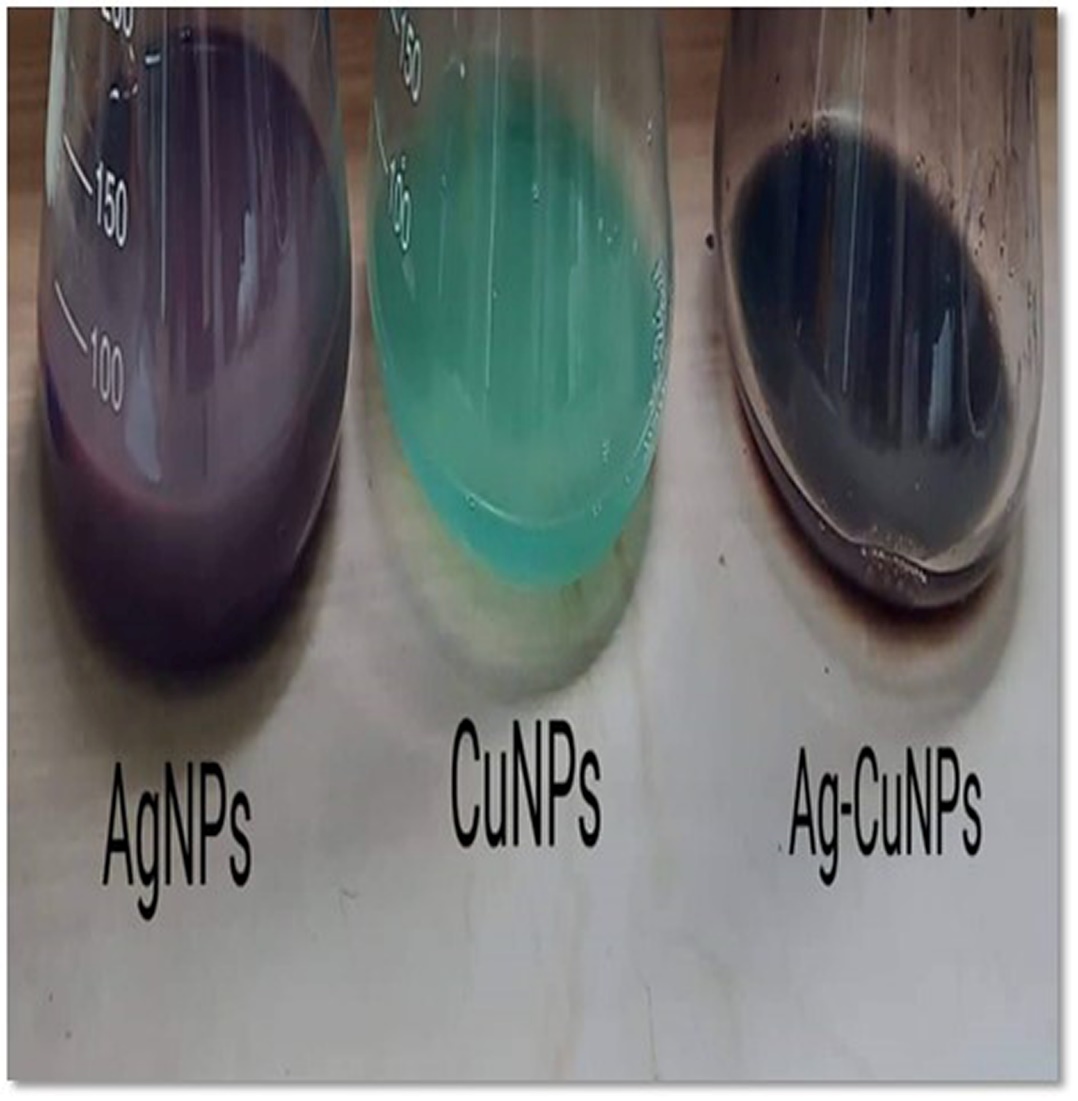

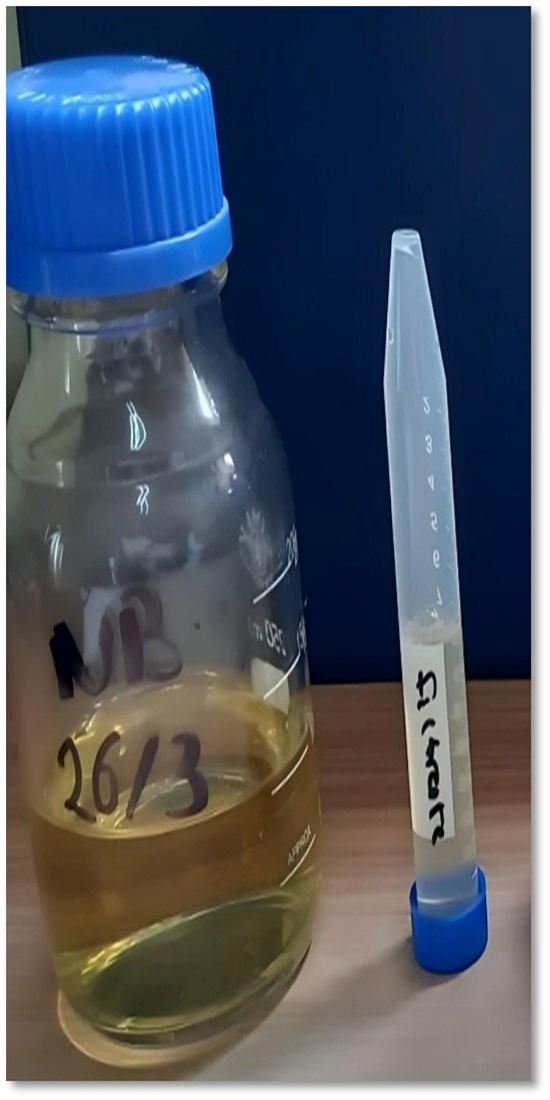


**Figure S1: Change in color suggesting biosynthesis of nanoparticles by *Staphylococcus* *aureus***

Supplement: Supplementary file 1 — Supplementary file1 (DOCX 284 kb) [file 12088_2024_1229_MOESM1_ESM.docx]

**
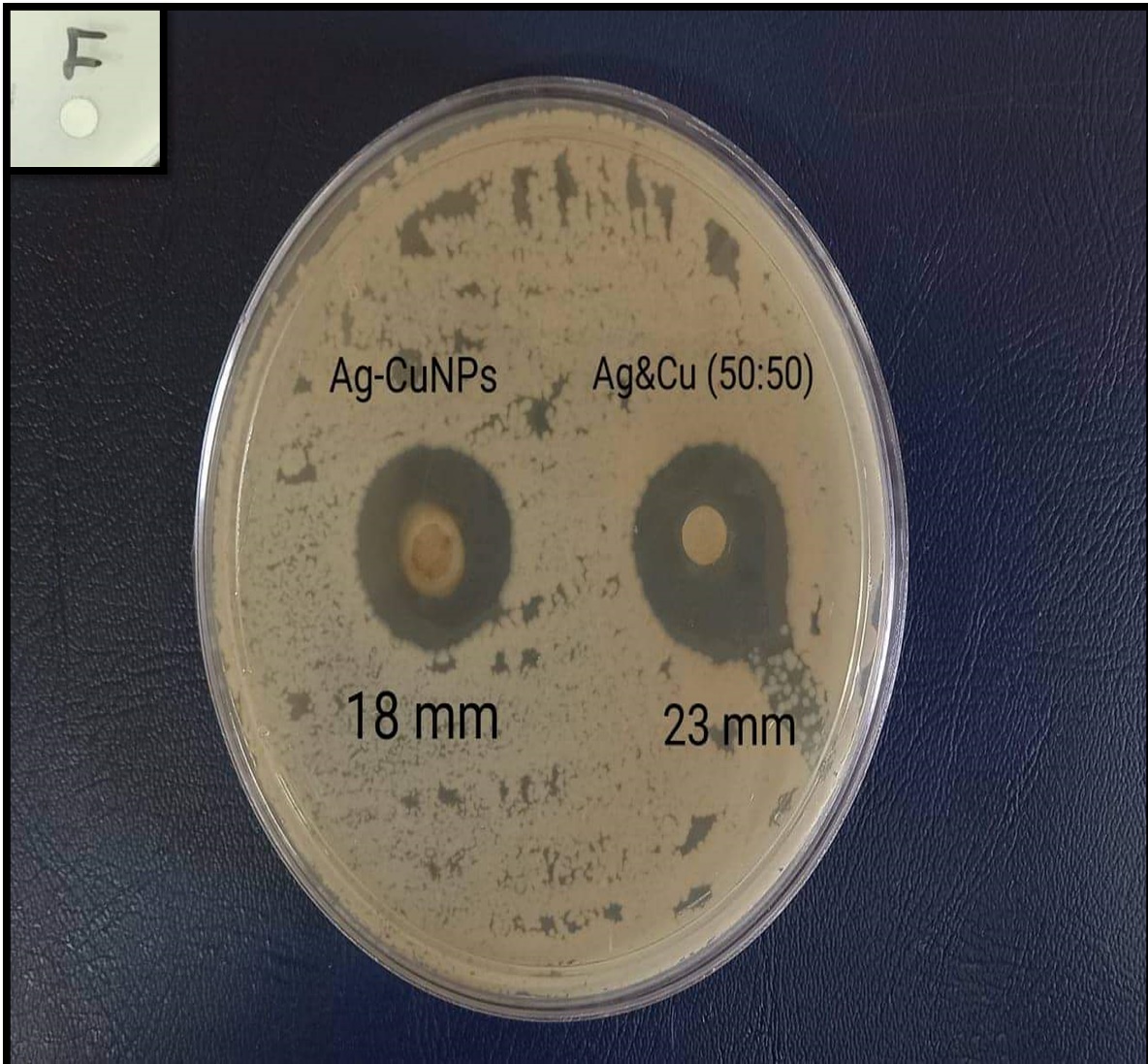
**

**Figure S2: Assayed Ag-Cu NPs, and Ag & Cu NPs on *B. cereus***

Supplement: Supplementary file 2 — Supplementary file2 (DOCX 393 kb) [file 12088_2024_1229_MOESM2_ESM.docx]
