# Supplementary material for: Biosynthesis of Silver, Copper, and Their Bi-metallic Combination of Nanocomposites by Staphylococcus aureus: Their Antimicrobial, Anticancer Activity, and Cytotoxicity Effect
Source: Indian J Microbiol. 2024 Mar 8;64(4):1721–37. doi: 10.1007/s12088-024-01229-2 (PMC11645382; doi:10.1007/s12088-024-01229-2)
Supplement: Supplementary file 3 — Supplementary file3 (DOCX 510 kb) [file 12088_2024_1229_MOESM3_ESM.docx]

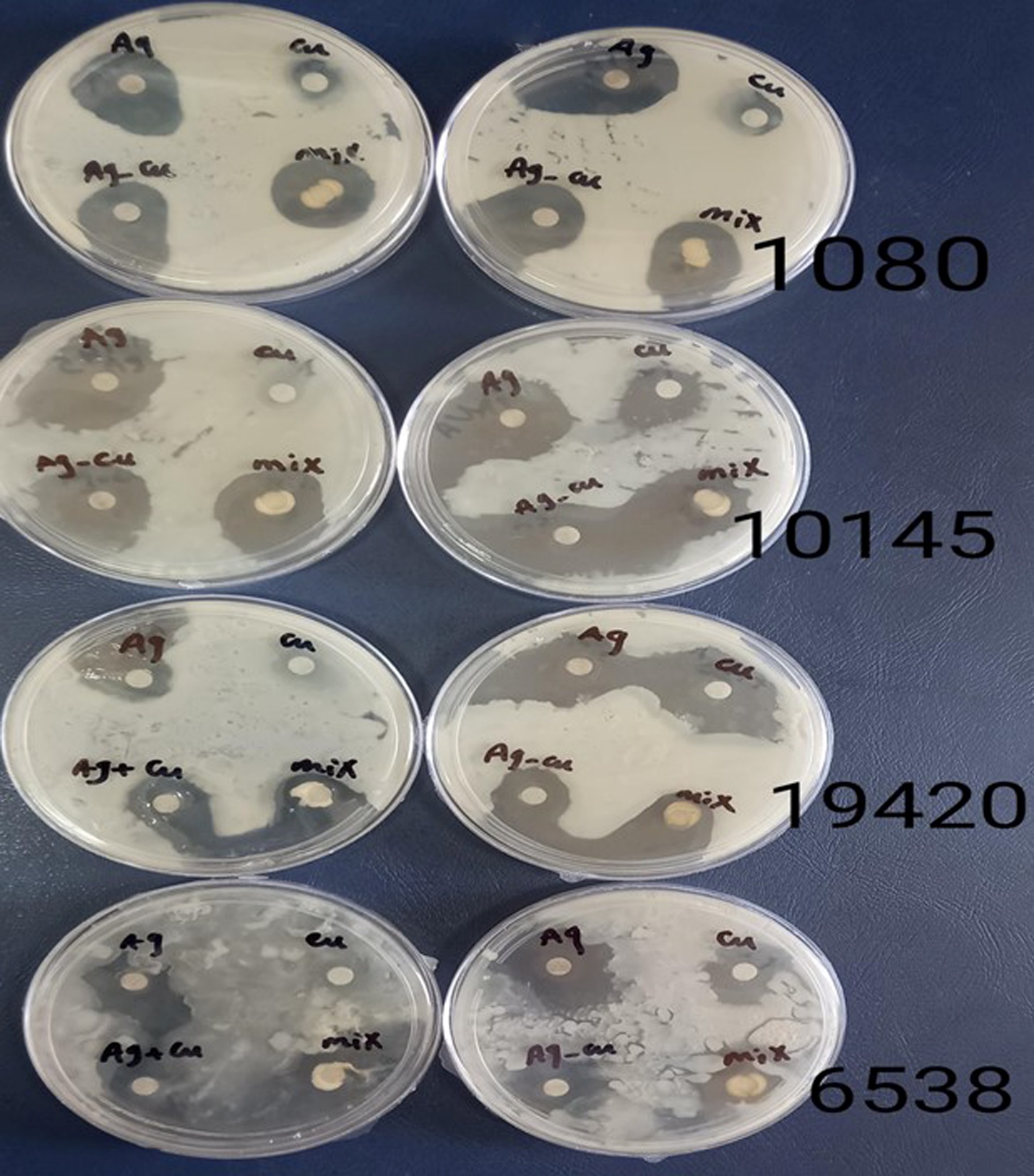


**Figure S3 : The antibacterial activity of the assayed nanoparticles, in which 1080 is *B. Cereus*, 10145 is *P. Aerginosa*, 19420 is *M. Smegamtis*, and 6538 is *S aureus.***
