# Supplementary material for: Biosynthesis of Silver, Copper, and Their Bi-metallic Combination of Nanocomposites by Staphylococcus aureus: Their Antimicrobial, Anticancer Activity, and Cytotoxicity Effect
Source: Indian J Microbiol. 2024 Mar 8;64(4):1721–37. doi: 10.1007/s12088-024-01229-2 (PMC11645382; doi:10.1007/s12088-024-01229-2)
Supplement: Supplementary file 4 — Supplementary file4 (DOCX 282 kb) [file 12088_2024_1229_MOESM4_ESM.docx]

**
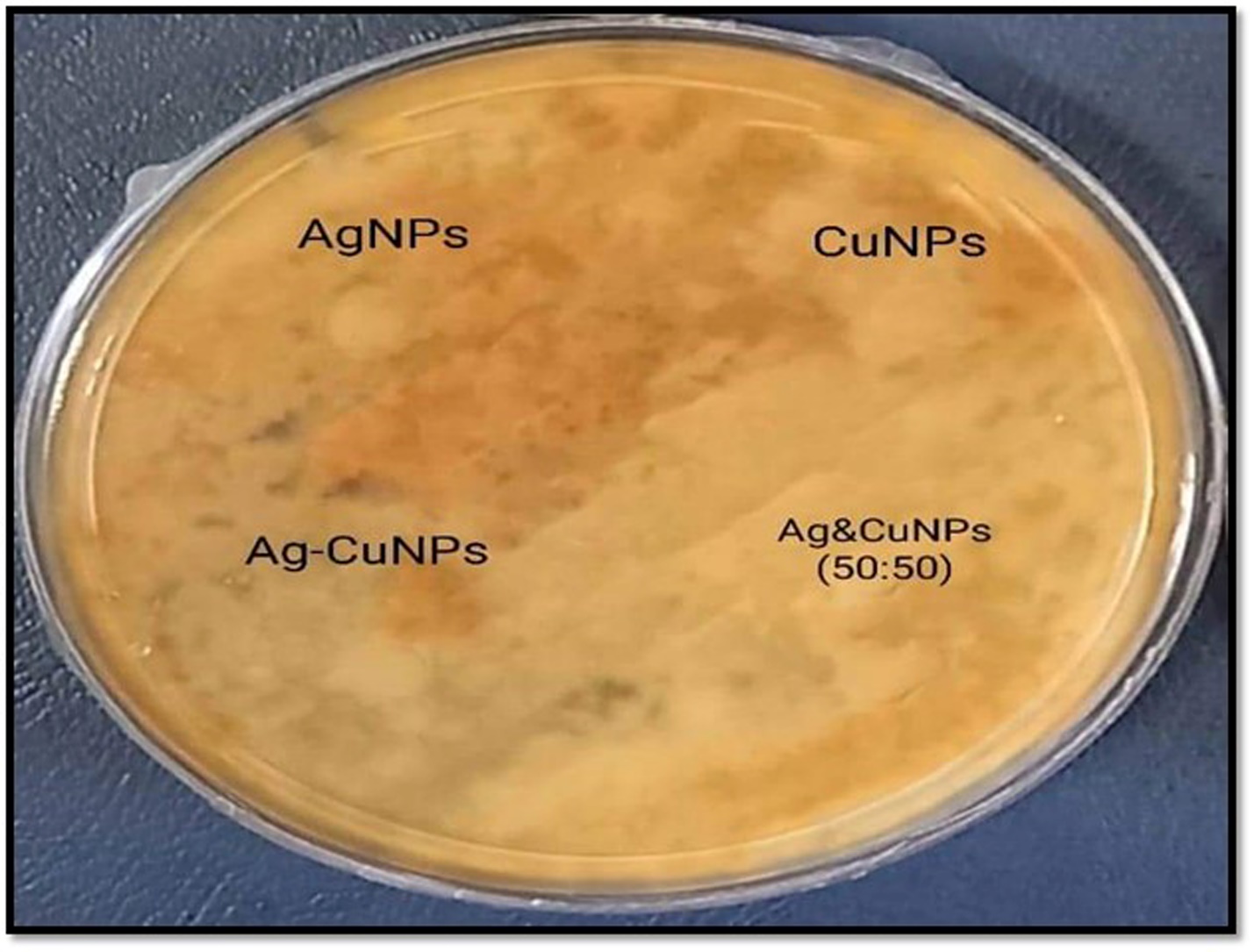
**

**Figure S4: A. fumigatus showing resistance against all the assayed biosynthesized nanoparticles**
