# Supplementary material for: Biosynthesis of Silver, Copper, and Their Bi-metallic Combination of Nanocomposites by Staphylococcus aureus: Their Antimicrobial, Anticancer Activity, and Cytotoxicity Effect
Source: Indian J Microbiol. 2024 Mar 8;64(4):1721–37. doi: 10.1007/s12088-024-01229-2 (PMC11645382; doi:10.1007/s12088-024-01229-2)
Supplement: Supplementary file 5 — Supplementary file5 (DOCX 542 kb) [file 12088_2024_1229_MOESM5_ESM.docx]

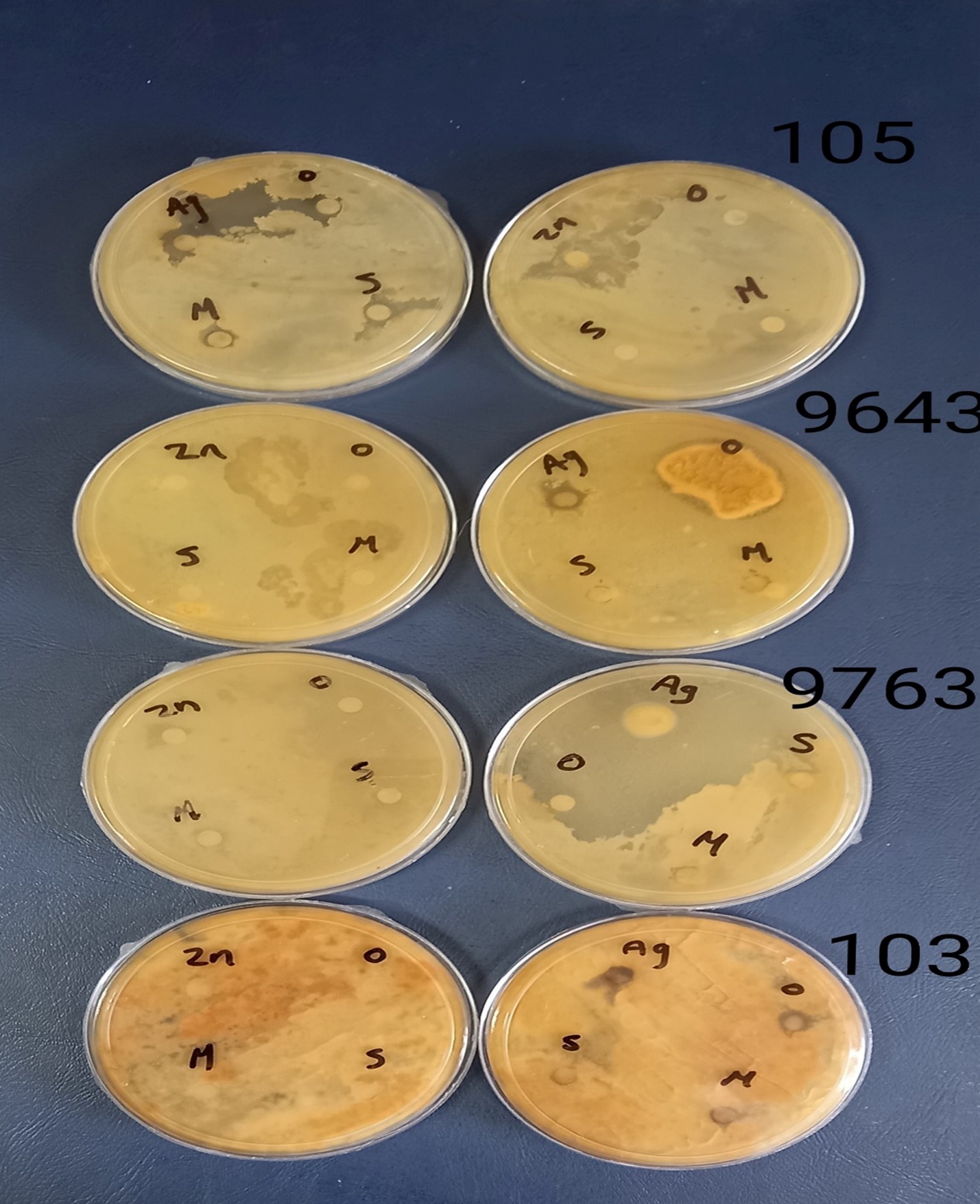


**Figure S5: The antifungal of the assayed nanoparticles in which, EMCC 105 is *C. Albicans*, ATCC 9643 is *A.flavus*, ATCC 9763 is *S. Cerevasie* , EMCC 103 is *A. Fumigamtis***
